# Comparison of the clinical frailty score (CFS) to the National Emergency Laparotomy Audit (NELA) risk calculator in all patients undergoing emergency laparotomy

**DOI:** 10.1111/codi.16089

**Published:** 2022-03-15

**Authors:** Subbra Palaniappan, Roy L. Soiza, Siobhan Duffy, Susan J. Moug, Phyo Kyaw Myint, the Older People’s Surgical Outcomes Collaborative (OPSOC), The Emergency Laparoscopic, Laparotomy Scottish Audit (ELLSA)

**Affiliations:** ^1^ 1019 University of Aberdeen Aberdeen UK; ^2^ Aberdeen Royal Infirmary NHS Grampian Aberdeen UK; ^3^ Royal Alexandra Hospital NHS Greater Glasgow & Clyde Paisley UK

**Keywords:** emergency laparotomy, frailty, NELA score, surgery

## Abstract

**Aim:**

There is evolving evidence that preoperative frailty predicts outcomes of older adults undergoing emergency laparotomy (EmLap). We assessed frailty scoring in an emergency surgical population that included patients of all ages and then compared this to an established perioperative prognostic score.

**Method:**

Data from the prospective Emergency Laparoscopic and Laparotomy Scottish Audit (ELLSA; November 2017–October 2018) was used. All adults over 18 were included. Frailty was measured using 7‐point clinical frailty score (CFS). Outcome measures: 30‐day mortality, hospital length of stay (LOS), 30‐day readmission. Areas under the receiver‐operating characteristic (ROC) curves were calculated for CFS (1–7) and compared to the National Emergency Laparotomy Audit (NELA) score with Forest plots used to compare 30‐day mortality across CFS and NELA categories.

**Results:**

A total of 2246 patients (median age 65 years [IQR 51–75]; female 51%) underwent EmLap (60% for colorectal pathology). A total of 10.6% were frail preoperatively (≥CFS 5). As CFS increased so did 30‐day mortality (2.1% CFS1 to 25.3% CFS6 and 7; *ꭓ* ^2^78.2, *p* < 0.001) and median LOS (10 days CFS1 to 20 days CFS6 and 7; *p* < 0.001). Readmission rates did not differ significantly across CFS. ROC (95% CI) for mortality was 0.71 (0.65–0.77) for CFS and 0.84 (0.78–0.89) for NELA. Addition of CFS to NELA did not increase ROC value.

**Conclusion:**

This study supports the prognostic role of frailty in the emergency surgical setting, finding increasing frailty to be associated with increased mortality and longer LOS in adults of all ages. Although NELA performed better, CFS remained predictive and has the advantage of being calculated preoperatively to aid decision‐making and treatment planning.

**What does this paper add to the literature?**

This is the first paper to show that frailty scoring is prognostic in adults of all ages undergoing emergency surgery. Although NELA score performs better, the clinical frailty score can be done preoperatively providing guidance for perioperative pathways and decision‐making in this high‐risk patient population.

## INTRODUCTION

Traditionally, clinical decision‐making in emergency surgery was guided by specific factors such as age, comorbidities and current clinical status and presentation. With none of these providing absolute clinical certainty for outcomes, risk‐scoring systems were developed including P‐POSSUM (Portsmouth Physiological and operative severity score for the enumeration of mortality and morbidity), that has now been replaced with the National Emergency Laparotomy Audit (NELA) risk calculator score [1, 2, 3, 4. Developed from the world's largest prospective emergency surgery database, the NELA risk calculator predicts 30‐day mortality from twenty pre‐ and intraoperative variables and is routinely collected across England and Wales.

Recently, frailty has been reported in the emergency surgical population [5, 6. Frailty is associated with increasing age and it can be defined as a “biological syndrome of decreased reserve and resistance to stressors, resulting from cumulative decline across multiple physiological systems and causing vulnerability to adverse outcomes [7]”. Using the clinical frailty score (CFS), the UK Emergency Laparotomy and Frailty (ELF) study reported that 20% of older adults ≥65 years undergoing emergency surgery were frail on admission [6, 8. Starting from a CFS of 1 (fit and healthy) and extending up to CFS 7 (severely frail), increasing frailty was found to predict mortality (30 and 90‐day), morbidity and discharge destination [6, 9. Although promising for clinical integration, the role of frailty has not been validated in another emergency surgical cohort, nor in younger adults where frailty has been previously reported uncertain. [5, 10

Our primary aim was to validate the prognostic role of frailty in a population undergoing emergency surgery that included adults of all ages. The secondary aim was to directly compare the CFS to the NELA risk calculator to assess CFS as a potential point‐of‐care prognosticator for mortality.

## METHODS

Prospective patient data was submitted locally from each of the 18 acute surgical units in Scotland from November 2017 to October 2018 to a central database as part of the prospective Emergency Laparotomy and Laparoscopic Scottish Audit (ELLSA). ELLSA forms part of a series of Scottish Government initiatives within the Modernising Patient Pathways Programme [11]. The current study was conceived, designed, and led by the Older Persons Surgical Outcomes Collaboration (https://www.opsoc.eu). NHS National Services Scotland Caldicott Guardian provided approval. Individual patient consent was not required as patient identifiable information from each site was anonymised before central data transmission to the authors.

Inclusion criteria were as per the ELLSA and NELA criteria as follows [11, 12:

- Patients aged 18 years and above
- Admitted and underwent expedited, urgent or emergency (National Confidential Enquiry into Patient Outcome and Death definitions) open laparotomy, laparoscopic or laparoscopically‐assisted abdominal procedures (referred to as EmLap)
- Operations on gastrointestinal tract where the major procedure was limited to stomach, small intestine, large intestine, rectum, intraperitoneal haematomas and abscesses, incarcerated hernias with bowel resection/repair or adhesiolysis, substantial abdominal wound dehiscence and returns to theatre for elective general surgery complications
- Laparotomy/laparoscopy with inoperable pathology where the intention was to perform a definitive procedure

Exclusion criteria included [11, 12:

- Patients under 18 years
- Purely diagnostic or elective laparotomy/laparoscopy
- Emergency hernia repair without bowel resection or division of adhesions
- All oesophageal, pancreatic, splenic, hepatobiliary, appendiceal, urological, vascular, organ transplant, trauma, obstetric or gynaecological operations

Baseline demographic, preoperative data and outcome data was recorded. This included sex, age, American Society of Anaesthesiologists physical status classification (ASA) with ASA ≥ 3 indicating at least severe systemic disease. [13]

CFS was classified as 1 = very fit, 2 = well, 3 = well with treated comorbid disease, 4 = apparently vulnerable, 5 = mildly frail, 6 = moderately frail and 7 = severely frail with CFS ≥ 5 considered frail [8]. Due to insufficient numbers of patients in CFS 6&7 categories, they were grouped together.

The NELA score was calculated by ELLSA auditors based on pre‐ and perioperative data. The percentage outcomes were stratified into low risk (<5%), intermediate risk (5%–10%) and high risk (>10%) as per the Royal College of Surgeons of England “The High‐Risk General Surgical Patient: Raising the Standard” report. [14]

Operative and post‐operative data was recorded by type of operation, postoperative care setting, total hospital length of stay (LOS), 30‐day readmission and 30‐day mortality.

### Statistical analysis

This was performed using IBM SPSS Statistics version 25.0. Age, NELA score, CFS (six categories: 1, 2, 3, 4, 5, 6 and 7) and LOS were reported using median and interquartile ranges (IQR). The remaining data was reported using numerical figures and percentages. Primary outcome measures were 30‐day mortality rate, total LOS and hospital readmission within 30 days. Specifically for LOS, the median for the entire cohort was calculated to allow LOS to be categorised as short or long LOS.

For secondary analysis, areas under the receiver operating characteristic (ROC) curves for the outcome of 30‐day mortality were calculated for CFS and NELA score to allow for direct performance comparison along with a Forest plot comparing 30‐day mortality between CFS and NELA using adjusted odds ratios with a 95% confidence interval. The proportions of younger (under 65 years) and older people with frailty (CFS 5 or above) were calculated, along with their respective ASA scores.

## RESULTS

### Basic demographics

The study population included 2,246 patients (median age 56 years [IQR 50–74]; female 51%) (Table 1) of whom 50.1% were aged 65 years and above, 10.6% were frail (CFS ≥ 5) and nearly 60% were ASA ≥ 3. Median CFS was 2 (IQR 2–3) and median NELA score was 3.7% (IQR 1.1–10.0). The mean ASA scores of older and younger patients in frail and nonfrail groups are shown in Table S1.

**TABLE 1:** Basic demographics of cohort and primary study outcomes

Basic demographics and outcomes (total cohort *n* = 2246)		
Sex (*n* = 2246)		Female – 1,147 (51%) and male – 1,099 (49%)
Age (*n* = 2245)		Median age 65 years (IQR 51–75) ≥65 years – 1,124 patients (50.1%)
ASA (*n* = 2157)	1 – normal healthy	9.0%
	2 – mild systemic disease	31.8%
	3 – severe systemic disease	39.0%
	4 – severe systemic disease that is constant threat to life	17.7%
	5 – moribund, not expected to survive without operation	2.4%
National Emergency Laparotomy Audit score (*n* = 1080)		Median score 3.7% (IQR 1.1–10) <5% (low risk) – 612, 56.6% 5–10% (intermediate risk) – 195, 18% >10% (high risk) – 273, 25.2%
Clinical frailty score (*n* = 1434)	Median CFS 2 [IQR2‐3]	
	1 (very fit)	330, 23.0%
	2 (well)	454, 31.7%
	3 (well with treated comorbid disease)	311, 21.7%
	4 (apparently vulnerable)	187, 13.0%
	5 (mildly frail)	77, 5.4%
	6 and 7 (moderately and severely frail)	75, 5.2%
Clinical frailty score by younger age (*n* = 1434)		<65 years of age
	1 (very fit)	274/330, 83.0%
	2 (well)	269/454, 59.3%
	3 (well with treated comorbid disease)	114/311, 36.7%
	4 (apparently vulnerable)	50/187, 26.7%
	5 (mildly frail)	18/77, 23.4%
	6 and 7 (moderately and severely frail)	20/75, 26.7%
Length of stay (*n* = 2067)		13.00 days (IQR 8.00–24.00)
30‐day mortality (*n* = 2244)		203 deceased, 9.0%
30‐day readmission (*n* = 2192)		1,965 not readmitted, 89.6% 158 readmitted to same specialty, 7.2% 69 readmitted to different specialty, 3.1%

Note

Total length of stay, 30‐day mortality and 30‐day readmission.

Abbreviation: ASA, American Society of Anaesthesiologists physical status classification.

Consistent with NELA [15], the most common types of surgery were small bowel resection (*n* = 374, 16.7%), right colectomy (*n* = 284, 12.6%), Hartmann's procedure (*n* = 256, 11.4%) and adhesiolysis (*n* = 253, 11.3%) (Table S2). Overall colorectal procedures accounted for 60%.

### Frailty and 30‐day mortality, length of stay and 30‐day readmission

Overall, 30‐day mortality for all patients was 9% (203/2244) (Table 1). As CFS increased so did the 30‐day mortality. Within CFS 1 the mortality rate was 2.1%, CFS 2 4.4%, CFS 3 10.3%, CFS 4 17.1%, CFS 5 15.6% and 25.3% within CFS 6&7 (*ꭓ* ^2^ 78.2, *p* < 0.001) (Table 2). Patients in CFS 6 and 7 had the highest risk of 30‐day mortality (odds ratio (OR) 15.7, 95% CI: 6.29–38.97, *p* < 0.001) compared with CFS 1. After adjusting for age, sex, ASA and antibiotic provision, patients in CFS 6 and 7 had an adjusted OR (aOR) of 3.62 (95% CI: 1.20–10.96, *p* = 0.023) (Table 3).

**TABLE 2:** Primary outcomes against CFS

Clinical frailty score	Total length of stay (*n* = 1326)	30‐day mortality (*n* = 1432)	30‐day readmission (*n* = 1402)
1	10.00 days (IQR 6.00–15.00)	7 deceased, 2.1%	293 not readmitted, 89.6% 25 readmitted to same specialty, 7.6% 9 readmitted to different specialty, 2.8%
2	12.00 days (IQR 8.00–20.00)	20 deceased, 4.4%	401 not readmitted, 90.5% 27 readmitted to same specialty, 6.1% 15 readmitted to different specialty, 3.4%
3	14.00 days (IQR 9.00–23.00)	32 deceased, 10.3%	264 not readmitted, 88.0% 27 readmitted to same specialty, 9.0% 9 readmitted to different specialty, 3.0%
4	17.00 days (IQR 10.75–28.00]=)	32 deceased, 17.1%	167 not readmitted, 90.8% 6 readmitted to same specialty, 3.3% 11 readmitted to different specialty, 6.0%
5	19.00 days (IQR 13.00–28.00)	12 deceased, 15.6%	72 not readmitted, 96.0% 2 readmitted to same specialty, 2.7% 1 readmitted to different specialty, 1.3%
6 and 7	20.00 days (IQR 12.00–32.00)	19 deceased, 25.3%	65 not readmitted, 89.0% 2 readmitted to same specialty, 2.7% 6 readmitted to different specialty, 8.2%
Statistical significance	*p* < 0.001	*ꭓ* ^2^ 78.2, *p* < 0.001	*p* > 0.05

Abbreviation: CFS, clinical frailty score.

**TABLE 3:** Crude odds ratio and adjusted odds ratio of the chance of 30‐day mortality, short versus long length of stay and 30‐day readmission rate, comparing increasing frailty versus patient defined as very fit using binary logistic regression

30 day‐mortality		Crude odds ratio (95% CI)	*p*‐value	Adjusted odds ratio^a^ (95% CI)	*p*‐value
CFS compared with patients very fit					
CFS	(1)	Reference	Reference	Reference	Reference
	2	2.14 (0.89–5.11)	0.088	1.13 (0.41–3.08)	0.816
	3	5.29 (2.30–12.18)	0.000	2.57 (0.96–6.92)	0.062
	4	9.53 (4.11–22.07)	0.000	3.28 (1.19–9.09)	0.022
	5	8.52 (3.23–22.46)	0.000	2.54 (0.81–7.96)	0.109
	6 and 7	15.7 (6.29–38.97)	0.000	3.62(1.20–10.96)	0.023

Total length of stay^b^		Crude odds ratio (95% CI)	*p*‐value	Adjusted odds ratio (95% CI)	*p*‐value
CFS compared with patients very fit					
CFS	(1)	Reference	Reference	Reference	Reference
	2	1.64 (1.22–2.22)	0.001	1.00 (0.70–1.42)	0.98
	3	2.31 (1.66–3.21)	0.000	1.07 (0.72–1.62)	0.73
	4	3.08 (2.08–4.56)	0.000	1.35 (0.82–2.20)	0.24
	5	4.78 (2.70–8.46)	0.000	1.70 (0.88–3.29)	0.12
	6&7	4.48 (2.52–7.97)	0.000	1.76 (0.89–3.46)	0.10

30‐day readmission		Crude odds ratio (95% CI)	*p*‐value	Adjusted odds ratio (95% CI)	*p*‐value
CFS compared with patients very fit					
CFS	(1)	Reference	Reference	Reference	Reference
	2	0.90 (0.56–1.45)	0.67	1.12 (0.64–1.95)	0.69
	3	1.18 (0.72–1.93)	0.53	1.68 (0.90–3.14)	0.10
	4	0.88 (0.48–1.62)	0.68	1.39 (0.64–3.03)	0.40
	5	0.36 (0.11–1.20)	0.10	0.43 (0.09–1.98)	0.28
	6 and 7	1.06 (0.47–2.40)	0.89	1.63 (0.61–4.34)	0.33

Abbreviations: CFS, clinical frailty score; CI, 95% confidence interval.

^a^ Adjusted by age linearly, sex (male, female), ASA (1, 2, 3, 4, 5) and sepsis antibiotic provision (not given antibiotics, given antibiotics).

^b^ Total length of stay coded as short (≤13.00 days) or long (>13.00 days).

Median LOS for entire cohort was 13 days (IQR 8.00–24.00) (Table 1). A higher median LOS was associated with an increasing CFS; 10 days (IQR 6–15) in CFS 1 to 20 days [IQR 12–32] in CFS 6 and 7 (*p* < 0.001) (Table 2). OR for CFS 6&7 compared to CFS 1 for LOS was 4.48 (95% CI: 2.52–7.97, *p* < 0.001) but when adjusted, attenuated to an OR of 1.76 (95% CI: 0.89–3.46; *p* = 0.10) (Table 3).

30‐day readmission rate was 10.3% but these rates did not differ significantly across CFS with 34 patients (0.03%) being readmitted in CFS 1 and eight patients (0.01%) being readmitted in CFS 6 and 7 (*p* > 0.05) (Tables 1 and 2).

### Comparison of CFS to NELA score in predicting 30‐day mortality

**FIGURE 1:**
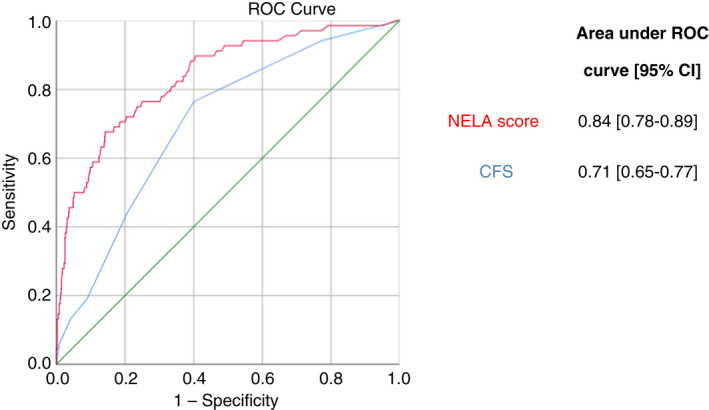
Receiver‐operating characteristic (ROC) curve – CFS and NELA versus 30‐day mortality (alive or dead). NELA is superior to CFS but CFS still has good predictive value. CFS, clinical frailty score; NELA, national emergency laparotomy audit; CI, 95% confidence interval

The areas under the receiver‐operating characteristic (ROC) curve in relation to 30‐day mortality with 95% CI for CFS and NELA were 0.71 (0.65–0.77) and 0.84 (0.78–0.89) respectively (Figure 1). Combining CFS and NELA did not increase the area under the curve.

**FIGURE 2:**
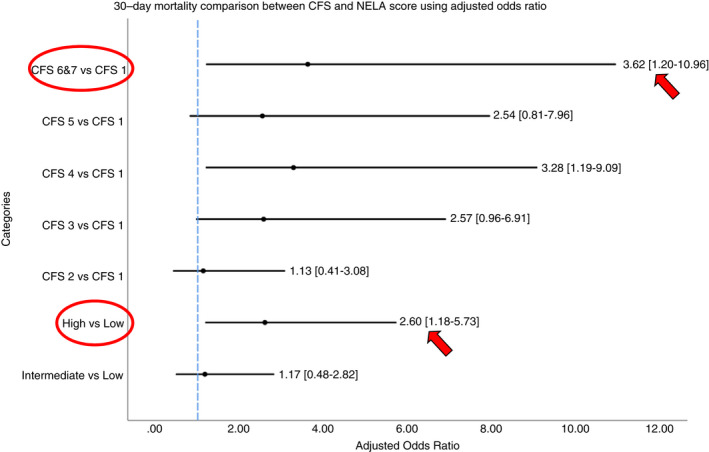
Forest plot comparing 30‐day mortality between CFS and NELA using adjusted odds ratio with 95% CI. CFS categorised into 1, 2, 3, 4, 5, 6 and 7. NELA score categorised using low, intermediate and high. CFS 1 and NELA low score used as reference categories. Blue hashed line is aOR of 1.00. Comparability between CFS 6 and 7 versus CFS 1 and NELA high versus low in predicting mortality based on adjusted odds ratio. CFS, clinical frailty score; CI, 95% confidence interval; NELA, National Emergency Laparotomy Audit

A Forest plot was charted to compare 30‐day mortality across varying levels of frailty by CFS (Figure 2) and NELA categories (Table 4). In the frailest patients (CFS 6 and 7 vs CFS 1) the aOR was 3.62 (95% CI: 1.20–10.96, *p* = 0.02), while the aOR for those with high versus low NELA scores was 2.60 (95% CI: 1.18–5.73, *p* = 0.02).

**TABLE 4:** Crude odds ratio and adjusted odds ratio of the chance of 30‐day mortality comparing increasing NELA score versus patient defined as low NELA score using binary logistic regression

30 day‐mortality		Crude odds ratio (95% CI)	*p*‐value	Adjusted odds ratio^a^ (95% CI)	*p*‐value
NELA score compared with low NELA score					
NELA score	Low	Reference	Reference	Reference	Reference
	Intermediate	3.28 (1.57–6.83)	0.002	1.17 (0.49–2.82)	0.72
	High	15.00 (8.42–26.74)	0.000	2.60 (1.18–5.73)	0.02

Abbreviations: CI, 95% confidence interval; NELA, National Emergency Laparotomy Audit.

^a^ Adjusted by age linearly, sex (male, female), ASA (1, 2, 3, 4, 5) and sepsis antibiotic provision (not given antibiotics, given antibiotics).

## DISCUSSION

This study validates the role of frailty in patients undergoing emergency laparotomy in that preoperative frailty score is associated with greater 30‐day mortality and longer total LOS [6]. By including all ages, not just older adults 65 years and above, this study confirms the existence of frailty in the younger emergency surgical population and that frailty negatively impacts on their clinical outcomes [5, 10. Younger and older patients with frailty had similar mean ASA scores. This reinforces the importance of frailty over age as a prognostic marker, though both are important. Comparison of the prognostic usefulness between CFS and the routinely applied NELA Score has not been previously performed and we report that CFS compares favourably to NELA for 30‐day mortality. Overall, with the clinical advantages of being free, easy to apply and most importantly, applicable preoperatively, frailty scoring should be routinely implemented into the acute adult surgical setting. [5, 6, 10

Preoperatively, the NELA score estimates its perioperative parameters, adding a degree of estimation and possible error. This means that the CFS carries significant advantage for surgeons and patients. By helping to correctly identify frail patients quickly, surgeons can then counsel patients and their families fully about risks, engage the appropriate frailty specific clinical pathway and liaise with appropriate allied health professionals to deliver the best, most appropriate, patient‐centred care. There may be settings where application of the CFS is not applicable, for example during the COVID‐19 pandemic, the National Institute for Health and Care Excellence (NICE) stated that CFS is not appropriate for clinical assessment of patients with stable long‐term disabilities, learning disabilities and autism^16^. However, NICE also excluded younger adults, an exclusion that the authors feel is challenged by this work.

Both the ELF and Hewitt et al. (2019) studies commented on the need for frailty scoring to be integrated into acute surgical assessment to help guide prognostication and decision‐making with the aim of developing frailty specific clinical pathways [5, 6. This message has also been consistently reiterated through NELA with their seventh report (2018–2019) stating that only 27.1% of frail patients over 65 years had geriatric specialist assessment and worryingly this figure continues to decline – 36.9% in 2017–2018 [15]. NELA are clear that the emergency laparotomy pathway must involve the expertise and input of geriatricians as part of the wider multidisciplinary team and this work supports such an approach. Evidence for improved outcomes as a result is reported by Vilches‐Moraga and Fox (2018) who developed a multidisciplinary pathway for frail general surgery patients [17]. Clinical outcomes are encouraging with a reduction in median LOS from 12.2 days to 9 days and fewer urgent medical reviews. This work focused on older adults, meaning further work is required to not only implement such pathways, but to consider including all frail patients, irrespective of age.

Study limitations include an insufficient number of patients to be able to categorise CFS 6 and 7 separately. The authors accept that other frailty scores could be applicable in the emergency setting and may allow improved categorisation. However, considering patients within these categories are the frailest, it is very likely that some of these patients within this group were considered futile and thus received palliative care and not be in ELLSA. Another limitation is that given the diverse nature of the UK population and globally, with frailty varying by country and ethnic group, there is an element of difficulty in directly applying the findings of this Scottish study from a 96% Caucasian base population to these groups [18, 19, 20. Cases with any missing covariates were excluded in statistical analysis, hence the final models had reduced sample size and thus prone to type II error which may explain some of the nonsignificant results observed such as 30‐day readmission. Finally, no data validation was performed on the individual data submitted by each local site.

## CONCLUSION

Frailty scoring preoperatively provides prognostic information that can be applied to all adults being considered for emergency surgery. Although the NELA score performed better, CFS has the clear advantage of being able to be applied rapidly in a time‐pressured situation. By virtue of its reliability, simple nature and quick prospective application, CFS can guide shared decision making between clinicians and patients.

## CONFLICT OF INTEREST

All of the authors declare no conflict of interests.

## AUTHOR CONTRIBUTIONS

All authors contributed to the concept, protocol, data analysis and drafting. All have reviewed the final versions.

## ETHICAL APPROVAL

Not applicable. Caldicott approval achieved.

## Supporting information

Table S1

Table S2

## Data Availability

All data is available directly from ELLSA.
