# Supplementary material for: Comparison of the clinical frailty score (CFS) to the National Emergency Laparotomy Audit (NELA) risk calculator in all patients undergoing emergency laparotomy
Source: Colorectal Dis. 2022 Mar 15;24(6):782–9. doi: 10.1111/codi.16089 (PMC9311201; doi:10.1111/codi.16089)
Supplement: Supplementary file 1 — Table S1 [file CODI-24-782-s001.docx]

Supplementary Table 1: Mean ASA scores in subgroups of young (<65y) and old (657 and above) categories and non-frail (CFS 1-3) and frail (CFS 4-7) groups.

| Patient category | Mean (SD) ASA score |
| --- | --- |
| Young, non-frail (N=642) | 2.28 (0.89) |
| Old, non-frail (N=426) | 2.80 (0.81) |
| Young, frail (N=83) | 3.29 (0.90) |
| Old, frail (N=243) | 3.33 (0.66) |
