# Supplementary material for: Comparison of the clinical frailty score (CFS) to the National Emergency Laparotomy Audit (NELA) risk calculator in all patients undergoing emergency laparotomy
Source: Colorectal Dis. 2022 Mar 15;24(6):782–9. doi: 10.1111/codi.16089 (PMC9311201; doi:10.1111/codi.16089)
Supplement: Supplementary file 2 — Table S2 [file CODI-24-782-s002.docx]

Supplementary Table 2: Types of operations – in descending order of frequency

| **Operation** | **Frequency** | **Percentage (%)** |
| --- | --- | --- |
| Small bowel resection | 374 | 16.7 |
| Colectomy: right (including ileocaecal resection) | 284 | 12.6 |
| Hartmann's procedure | 256 | 11.4 |
| Adhesiolysis | 253 | 11.3 |
| Colectomy: subtotal or pan proctocolectomy | 136 | 6.1 |
| Peptic ulcer suture or repair of perforation | 136 | 6.1 |
| Other | 115 | 5.1 |
| Stoma formation | 115 | 5.1 |
| Washout only | 85 | 3.8 |
| Colectomy: left (including anterior resection) | 76 | 3.4 |
| Exploratory/relook laparotomy only | 63 | 2.8 |
| Colorectal resection - other | 50 | 2.2 |
| Repair of intestinal perforation | 39 | 1.7 |
| Gastric surgery - other | 30 | 1.3 |
| Abdominal wall closure | 25 | 1.1 |
| Drainage of abscess/collection | 23 | 1.0 |
| Repair or revision of anastomosis | 23 | 1.0 |
| Enterotomy | 19 | 0.8 |
| Stoma revision | 17 | 0.8 |
| Laparostomy formation | 15 | 0.7 |
| Reduction of volvulus | 15 | 0.7 |
| Haemostasis | 12 | 0.5 |
| Gastrectomy - partial or total | 11 | 0.5 |
| Not stated | 11 | 0.5 |
| Intestinal bypass | 9 | 0.4 |
| Removal of foreign body | 9 | 0.4 |
| Resection of Meckel's diverticulum | 9 | 0.4 |
| Resection of other intra-abdominal tumour(s) | 9 | 0.4 |
| Abdominal wall reconstruction | 8 | 0.4 |
| Peptic ulcer - oversew of bleed | 8 | 0.4 |
| Debridement | 5 | 0.2 |
| Evacuation of haematoma | 4 | 0.2 |
| Repair of intestinal fistula | 2 | 0.1 |
